# Defining metrics for whole-genome sequence analysis of MRSA in clinical practice

**DOI:** 10.1099/mgen.0.000354

**Published:** 2020-03-31

**Authors:** Kathy E. Raven, Beth Blane, Narender Kumar, Danielle Leek, Eugene Bragin, Francesc Coll, Julian Parkhill, Sharon J. Peacock

**Affiliations:** ^1^​ Department of Medicine, University of Cambridge, Box 157 Addenbrooke’s Hospital, Hills Road, Cambridge, CB2 0QQ, UK; ^2^​ Next Gen Diagnostics LLC (NGD), Mountain View, CA and Wellcome Genome Campus, Hinxton, Cambridge, UK; ^3^​ London School of Hygiene & Tropical Medicine, Keppel Street, London, WC1E 7HT, UK; ^4^​ Department of Veterinary Medicine, University of Cambridge, Madingley Road, Cambridge, CB3 0ES, UK; ^5^​ Wellcome Sanger Institute, Wellcome Genome Campus, Hinxton, Cambridge, CB10 1SA, UK

**Keywords:** whole-genome sequencing, quality metrics, MRSA, translational

## Abstract

Bacterial sequencing will become increasingly adopted in routine microbiology laboratories. Here, we report the findings of a technical evaluation of almost 800 clinical methicillin-resistant *Staphylococcus aureus* (MRSA) isolates, in which we sought to define key quality metrics to support MRSA sequencing in clinical practice. We evaluated the accuracy of mapping to a generic reference versus clonal complex (CC)-specific mapping, which is more computationally challenging. Focusing on isolates that were genetically related (<50 single nucleotide polymorphisms (SNPs)) and belonged to prevalent sequence types, concordance between these methods was 99.5 %. We use MRSA MPROS0386 to control for base calling accuracy by the sequencer, and used multiple repeat sequences of the control to define a permitted range of SNPs different to the mapping reference for this control (equating to 3 standard deviations from the mean). Repeat sequences of the control were also used to demonstrate that SNP calling was most accurate across differing coverage depths (above 35×, the lowest depth in our study) when the depth required to call a SNP as present was at least 4−8×. Using 786 MRSA sequences, we defined a robust measure for *mec* gene detection to reduce false-positives arising from contamination, which was no greater than 2 standard deviations below the average depth of coverage across the genome. Sequencing from bacteria harvested from clinical plates runs an increased risk of contamination with the same or different species, and we defined a cut-off of 30 heterozygous sites >50 bp apart to identify same-species contamination for MRSA. These metrics were combined into a quality-control (QC) flowchart to determine whether sequence runs and individual clinical isolates passed QC, which could be adapted by future automated analysis systems to enable rapid hands-off sequence analysis by clinical laboratories.

## Data Summary

Sequence data generated during this study have been deposited in the European Nucleotide Archive under the accession numbers listed in Table S2 (available in the online version of this article) (https://www.ebi.ac.uk/ena).

**Impact Statement**

Bacterial whole-genome sequencing is a highly discriminatory technique that could be a powerful adjunct for infection control teams. This allows isolates (patients) to be identified as being part of an outbreak, enabling early action to prevent further dissemination. As importantly, isolates (patients) can be shown not to be linked with a suspected outbreak, preventing unnecessary infection control actions. Methicillin-resistant *Staphylococcus aureus* (MRSA) is a major cause of hospital infections and causes outbreaks both in hospitals and the community. Whole-genome sequencing of MRSA can now be performed within 24 h, and tools are under development to rapidly analyse the resulting data without the need for expert input, but quality-control (QC) metrics are required for this tool to be used in clinical practice. In this study we defined a number of QC metrics to support MRSA sequencing in clinical practice, culminating in a QC flowchart that could be adopted or adapted by future users.

## Introduction

Research evidence for the practical utility of MRSA sequencing as an adjunct to infection control practice supports its introduction into routine use. This includes the demonstration that sequencing is superior to other typing methods in discriminating between isolates of the same lineage [1–4], and that the combination of standard outbreak investigation and MRSA sequencing is superior to standard outbreak investigation alone [5]. Sequencing has been shown to exclude outbreaks in instances where patient clusters have occurred by chance [6], which could prevent unnecessary use of infection control resources. Furthermore, proactive use of MRSA sequencing has been proposed as part of a redesign in infection control practice [7], replacing the reactive use of typing as a late adjunct during established outbreak investigations. Support for this ‘Sequence First’ approach comes from a prospective study of genomic MRSA surveillance over 12 months at a clinical microbiology laboratory, in which 2282 MRSA from 1465 people were sequenced [5]. An integrated genomic and epidemiological analysis identified 173 separate transmission clusters containing between 2 and 44 cases and involving 598 people that were not detected by conventional infection control approaches. Economic cost-effectiveness studies for proactive MRSA sequencing are lacking in the published literature but are on-going.

There has been considerable debate about whether pathogen sequencing should be performed in a centralized facility or distributed throughout the network of hospital-based diagnostic laboratories. However, the pace at which sequencing instruments are being developed for clinical use means that sequencing will become increasingly feasible in any setting provided that robust methods are available to support high-quality real-time sequencing, combined with the availability of fully automated tools that undertake relatedness comparisons and provide interpretation guidelines for use of this information to healthcare workers without informatics expertise. This would support the rapid use of data to direct and optimize MRSA outbreak investigations. Progress has been made to describe the sequencing of MRSA within a 24 h time window (from the start of DNA extraction to sequence data generation) [8], which includes sequencing of colony picks directly from primary clinical culture plates [9]. Furthermore, a pilot evaluation of a commercial fully automated interpretation tool indicates that genetic relatedness can be generated within 90 s per isolate [10].

Here, we report the findings of a technical evaluation of almost 800 MRSA isolates that set out to define several key metrics that could support the analysis of MRSA sequence data in clinical practice. This includes the selection of mapping reference for relatedness determination using core-genome SNPs, the sequencing depth required to call a SNP, and defining cut-offs for the detection of *mec* genes and same-species contamination. These culminated in a quality-control (QC) metric flowchart to guide future adopters.

## Methods

### Study setting, patients and sample identification

The study was conducted at the Clinical Microbiology and Public Health Laboratory at the Cambridge University Hospitals NHS Foundation Trust (CUH), UK, under ethical approval from the National Research Ethics Service (ref: 11/EE/0499) and the Cambridge University Hospitals NHS Foundation Trust Research and Development Department (ref: A092428). Consecutive patients with samples that were MRSA positive were identified between 24 January and 1 November 2018 using the hospital IT system [EPIC EMR (Hyperspace 2014; Epic Systems Corporation)]. One MRSA isolate from each patient (generally the first available) was sought for sequencing, together with all isolates cultured from blood cultures. In the first 3 months of the study (24 January to 3 April), this was achieved by isolate retrieval from the frozen archive to provide look-back data for the subsequent 6-month prospective collection. Of the 147 cases identified with MRSA-positive samples in the first 3 months, 124 (84 %) patients had a stored MRSA isolate. Thereafter through to the end of the study, MRSA-positive cultures were retrieved in real-time from the clinical laboratory by the research team. Samples were flagged by laboratory staff and the culture plate retrieved on the same day, where possible. A total of 772 patients were identified as having MRSA-positive samples during this second period, of which 673 patients (87 %) had a clinical culture retrieved from which MRSA was obtained and sequenced. After de-duplication of cases presenting in the first and second periods, a total of 789 isolates from 784 patients were processed for sequencing. Samples were renumbered with an anonymous study code upon entry into the study.

### Microbiology

Freezer archive samples were plated onto Columbia Blood Agar (CBA) using a 1 µl loop and incubated overnight at 37 °C in air. A single colony was selected for sequencing, and a 10 µl loopful was stored at −80 °C in Microbank vials (Pro-lab Diagnostics). Plates that appeared contaminated were re-subbed onto CBA and incubated at 37 °C overnight prior to sequencing and re-storage. For prospectively obtained clinical samples, putative *S. aureus* was confirmed using the Staph Latex kit (Pro-lab Diagnostics). A single 2–3 mm colony was picked from clinical culture plates using a 1 µl loop and inoculated onto CBA and incubated overnight at 37 °C in air. Where colonies were smaller than 2 mm, several colonies were picked. Where bacterial growth was confluent (growth covering the majority or all of the agar plate), a 1 µl loopful was taken. If there were several positive plates for one clinical sample, the plate with the least visible background contamination was selected. As with the freezer archive, a single colony was selected for sequencing, and a 10 µl loopful was stored at −80 °C in Microbank vials.

### Sequencing and data analysis

Clinical MRSA isolates were sequenced in batches of 21 isolates plus three controls (described below). DNA extraction was performed using the QIAgen QIAamp DNA Mini extraction kit with the protocol amendments described previously [8]. Library preparation was performed using the Illumina Nextera DNA Flex kit with the protocol amendments described previously [8]. Isolates were sequenced on the Illumina MiniSeq with a run time of 13 h using the high output 150 cycle MiniSeq cartridge and the Generate Fastq workflow. Data were saved to an external hard drive. Species identification was performed using Kraken version 1 (https://ccb.jhu.edu/software/kraken/) with the miniKraken database (https://ccb.jhu.edu/software/kraken/dl/minikraken_20171019_8GB.tgz). Multilocus sequence type (ST) was identified using Ariba (https://github.com/sanger-pathogens/ariba/wiki/MLST-calling-with_ARIBA). Clonal complexes were determined based on assignments reported previously [5] or based on the allelic profile, allowing up to two alleles different to the reference ST. The presence of *mecA* (accession number HE681097, position 2790560 : 2792566), *mecB* (accession number AP009486, position 25 508–27 532) or *mecC* (accession number FR821779, position 35 681–37 678) was identified using Ariba (https://github.com/sanger-pathogens/ariba). Fastq files were mapped to clonal-complex (CC) specific references using smalt (https://www.sanger.ac.uk/science/tools/smalt-0), as described previously [8], for all isolates that were assigned to a ST that contained more than ten clinical isolates. CC-specific references were CC1 MW2 (accession number BA000033), CC5 N315 (accession number BA000018), CC8 USA300 (accession number CP000255), CC22 EMRSA15 (accession number HE681097), CC30 MRSA252 (accession number BX571856), CC45 CA347 (accession number CP006044) and CC59 M013 (accession number CP003166). In addition, all isolates were mapped to the CC22 EMRSA15 mapping reference. Variant calling and generation of the consensus fasta files were performed using Samtools and bcftools. Mobile genetic elements were removed from the alignment using the files available at http://figshare.com/authors/Francesc_Coll/5727779 and the script available in Github (https://github.com/sanger-pathogens/remove_blocks_from_aln.py). SNPs were identified using the script available in Github (https://github.com/sanger-pathogens/snp_sites). Pairwise differences in SNPs between isolate pairs was determined using the script available at https://github.com/simonrharris/pairwise_difference_count. Heterozygous SNPs (hetSNPs) were evaluated based on mapping to the CC22 reference and were identified as SNPs with <90 % support for a single base. The number of hetSNPs at least 50 bp apart was determined using the script available at Github (https://github.com/kumarnaren/mecA-HetSites-calculator), which required at least one read to be mapped in either orientation. The average coverage across the mapping reference, the standard deviation in coverage, and the depth of coverage for the *mec* gene of each isolate was determined using the same script. We downloaded genome sequence data for MRSA MPROS0386 generated previously over nine independent sequencing runs [8] from the European Nucleotide Archive, which were used for the analysis of variation in SNPs in the positive control.

### Contamination experiment

Detection of contamination with the same species was determined through a deliberate contamination experiment. MPROS1839 and MPROS2264 (both ST22s) were spiked with 0, 0.01, 0.1, 1, 10 and 20% concentration of DNA from MPROS0386 (ST22, ~100 SNPs from MPROS1839 and MPROS2264) and the resulting DNA combination sequenced as above.

### QC metrics

Each sequencing run contained 21 clinical MRSA isolates and three controls [positive control (MRSA MPROS0386), negative control (* Escherichia coli* NCTC12241) and a no-template control] with the aim of producing 50× of data for each isolate [8]. The controls had to pass the following QC metrics for the sequence run to pass QC: no-template control must have <95 000 reads (equating to <1 % contamination) matching to a species in Kraken [8]; negative control must have its best match to *E. coli* in Kraken, have <0.4 % of reads (equating to <1 % contamination) in Kraken matching to another species [8], have no *S. aureus* ST identified, and have no *mec* gene identified; positive control must have its best match to *S. aureus* in Kraken, have <0.4 % (equating to <1 % contamination) of reads in Kraken matching to another species [8], be identified as ST22, positive for *mecA*, and have at least 80 % of the CC22 mapping reference genome covered with at least 20× depth. Two sequence runs failed QC based on low output of data for the positive control (<20× depth and <80 % of the reference covered) and were repeated (Table S1). The positive control strain is available upon request.

All clinical isolates had to pass the following QC metrics: have their best match to *S. aureus* in Kraken, be positive for *mecA*/*mecB*/*mecC*, have <4 % reads (equating to <10 % contamination) in Kraken matching to another species [8], and have at least 80 % of the CC22 mapping reference genome covered at least 20× depth. Of the 789 isolates from 784 patients in the study collection, 25 failed these QC metrics the first time due to incorrect species [*n*=3; *Corynebacterium striatum* (*n*=1), *Staphylococcus haemolyticus* (*n*=2)]; contamination with a different species [*n*=7; *S. haemolyticus* (*n*=4), *Staphylococcus capitis* (*n*=1)]; no *mec* gene (*n*=4);<20× depth (*n*=10); or <80 % of the reference covered at 20× depth (*n*=5). After re-purification of the original culture to re-isolate MRSA, 22 of 25 isolates were successfully re-sequenced, leaving a total of 786 isolates that were included in the analysis (Table S2). Of the 25 isolates failing the initial QC metrics, 24 were from the prospective collection and one was from the retrospective archives. Of the five isolates failing QC due to heterozygous sites, all five were from prospective collection.

### Applicability to alternative settings

To determine the applicability of CC22 mapping to alternative clinical settings, raw fastq files were downloaded from the European Nucleotide Archive that were submitted by two studies that have been described previously [11, 12]. The first study collection consisted of 425 MRSA isolates associated with bloodstream infection in patients from across the UK between 2012–2013. Two clinical isolates failed our QC metrics (based on lack of *mec* gene detection) and were excluded, leaving 423 isolates for further analysis. The second collection consisted of 742 clinical isolates from two NHS hospital groups and a general district hospital in South-East London between 2011 and 2012. A total of 152 isolates failed our QC metrics for the following reasons: not being best matched to *S. aureus* in Kraken (*n*=8), >4 % of reads in Kraken matching to another species (*n*=80), no *mec* gene detected (*n*=54), or <20× depth of coverage (*n*=10) leaving a total of 590 isolates for further analysis. These were mapped to the CC22 reference and CC-specific mapping references as described above, with AUS0325 (accession number LT615218) used as the mapping reference for CC88.

## Results

### Overview of patients and isolates

We sequenced 786 MRSA isolates from samples submitted to the clinical laboratory from 782 patients. The majority of patients had isolates submitted from Cambridge University Hospitals (*n*=485), the remainder being from patients at two other hospitals in the locality (hospital A and hospital B) (112 and 44 cases, respectively) and GP surgeries (*n*=141). The majority of isolates were from multisite screens (528/786; 67 %), with the remainder from clinical samples including seven isolates (1 %) grown from blood cultures taken from six patients. *In silico* MLST from the sequence data demonstrated that the most common STs were ST22 (*n*=368, 47 %), followed by ST45 (*n*=81, 10 %) and ST59 (*n*=62, 8 %) with a total of 66 STs including 15 novel STs identified (Table S2). A total of 781 isolates were *mecA* (99 %) with the remaining five isolates containing *mecC*.

### CC-specific versus a single mapping reference

**Fig. 1.**
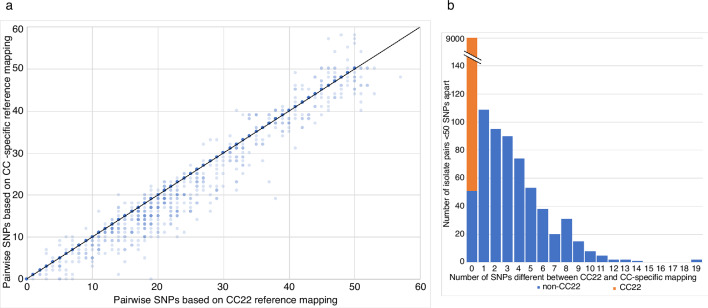
CC-specific versus a single mapping reference. (a) Graph showing the relationship between pairwise SNP distances based on mapping to a CC22 reference and mapping to a CC-specific reference, for all isolate pairs <50 SNPs apart that belonged to STs with >10 isolates in the collection, based on either method. Line indicates an exact match between the two methods. (b) Graph showing the number of SNPs different between CC22 and CC-specific mapping based on isolate pairs belonging to CC22 (orange) or non-CC22 (blue) STs.

We evaluated whether a single reference could be used for mapping, rather than the common practice of using CC-specific references [11, 13]. We based the analysis on isolates that were assigned to STs containing more than ten isolates (an arbitrary cut-off) (*n*=655, 83 %). The single CC22 reference used represented the dominant clonal complex in the collection and was ~46 000 and 53 000 SNPs from the next most common STs in the collection (ST59 and ST45). Fig. 1 shows a comparison of isolates that were within 50 SNPs of each other using a CC22 reference alone or a CC-specific mapping reference (*n*=399 isolates). There was a positive association between CC-specific versus CC22 mapping, with a median of 0 SNPs different (range 0–19) between the two approaches across all 399 isolates (9597 pairs), and a median of three SNPs different (range 0–19) for the 602 non-CC22 pairs (Fig. 1). We have reported elsewhere that a SNP cut-off of 25 SNPs robustly captures transmission events (Coll *et al*., under review). Based on binary categories of related (0–25 SNPs) or unrelated (>26 SNPs) for CC22 mapping, there were 44 pairwise discords (0.46 % of the total of 9632). A single ST59 pair was 25 SNPs apart based on CC22 mapping but 27 SNPs apart based on CC-specific mapping, and 43 isolate pairs were >25 SNPs (26–38 SNPs) apart based on CC22 mapping but <25 SNPs (14–24 SNPs) apart based on CC-specific mapping (Fig. S1). This provides a concordance of 93.6 % for non-CC22 isolate pairs and 99.5 % for all isolate pairs.

We repeated the analysis based on CC22 and CC-specific mapping in which we compared only a single randomly chosen genome that was <50 SNPs different for each isolate. This resulted in a median of 0 SNPs different (range 0–19 SNPs) with 65 % of the comparisons (total of 399 comparisons) comprising CC22 isolates. There were 9 discrepancies (2 %), with higher numbers of SNPs called using the CC22 mapping method. This provides a concordance of 93.6 % for non-CC22 isolate pairs and 98 % for all isolate pairs.

To determine whether the results were comparable with a different mapping reference we repeated the analysis using a generic CC30 reference. A total of 17 isolates (2 %) in the collection belonged to ST30. This demonstrated a median of 3 SNPs (range 0–24 SNPs) different between CC30 and CC-specific mapping across the isolate pairs (Fig. S2). Based on a 25 SNP cut-off, 1046 isolate pairs (10.8 %) were discrepant, providing an overall concordance of 89.2 %.

To investigate whether these findings are applicable to other clinical settings, we repeated the analysis using two other MRSA collections that have been described previously [11, 12], drawn from across the UK and from South-East London, respectively. The ST profiles of each collection are shown in Fig. S3. The analysis was based on isolates assigned to STs containing >5 isolates for the UK collection (*n*=362, 86 %), and >10 isolates for the London collection (*n*=500, 85 %), and focused on isolate genomes that were <50 SNPs different from another genome. When comparing CC22 and CC-specific mapping, there was a median of 0 SNPs (range 0–9 SNPs) and 0 SNPs (range 0–25 SNPs) different for all isolates and a median of 2 SNPs (range 0–9 SNPs) and 4 SNPs (range 0–25 SNPs) different for non-CC22 isolates, based on the UK and London collections, respectively. Based on binary categories of related (0–25 SNPs) or unrelated (>26 SNPs), there were 37 pairs of related isolates in the national collection (of which 27 were ST22 pairs), with 100 % concordance between CC22 and CC-specific mapping, and 1196 pairs of related isolates in the regional collection (of which 1118 were ST22), with 19 discrepancies between CC22 and CC-specific mapping. All 19 discrepancies in the regional collection belonged to ST8 (*n*=3) or ST36 (*n*=16), accounting for 21 and 36 % of related pairs and 0.6 and 1.1 % of all pairs in these STs, respectively. For the national and regional collections, respectively, this provided an overall concordance rate of 100 and 75.6 % for non-CC22 isolate pairs and 100 and 98.4 % for all isolate pairs.

### Defining the depth required to call a SNP

**Fig. 2.**
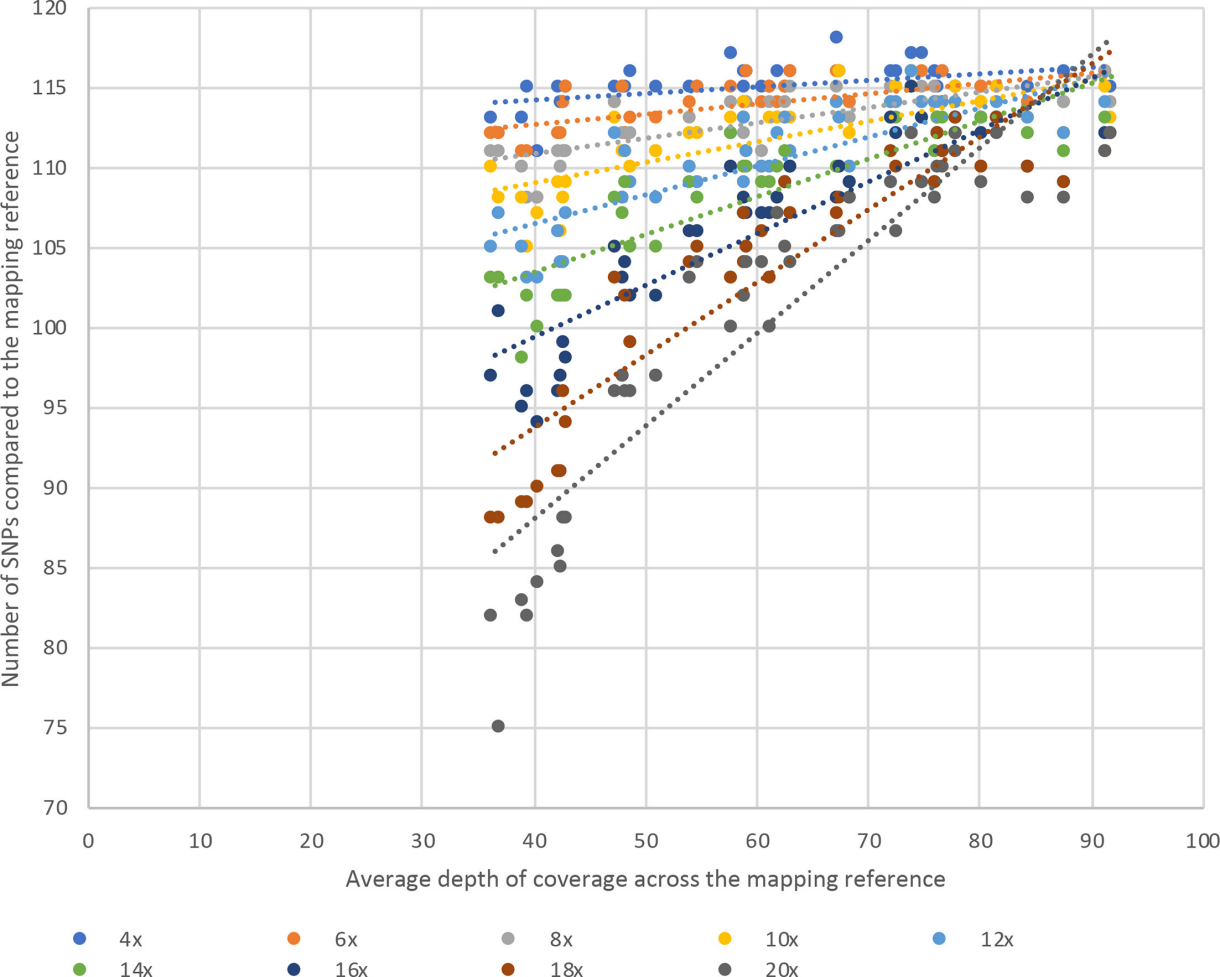
Sequencing depth to call a SNP as present. Graph showing the number of SNPs identified against the HO 5096 0412 mapping reference with MGEs removed (*y*-axis) in 43 sequencing replicates of MPROS0386 with different average depths of coverage (*x*-axis) based on varying thresholds for the depth of coverage required to call a SNP as present.

We explored the extent to which the number of called SNPs varied at different thresholds for the minimum depth of coverage required to call a base as present (as opposed to an unknown base call, which would not count towards the SNP count). This was evaluated over incremental 2× steps between 4× (which has been used previously [5]) and 20× using data from 43 independent sequencing runs of the MRSA control after mapping to a CC22 reference. The mean SNP number identified decreased as the threshold required to call a base as present increased (Fig. 2), with 101 SNPs at 20× rising to 115 SNPs at 4×. The SNP range across the 43 runs was highest at a 20× threshold for calling a base as present (37 SNPs) and lowest at a 4× threshold for calling a base as present (8 SNPs) (Table S3). There was comparable SNP calling at 4× (mean 115, range 8 SNPs), 6× (mean 114, range 9 SNPs) and 8× (mean 113, range 9 SNPs) at a base, regardless of the mean depth across the genome (Table S3).

The number of SNPs identified in the sequences with the highest mean depth of coverage across the genome would be predicted to be the true number of SNPs, on the basis that these have the most data to support base-calling. This is supported by findings that the SNP range was smallest at the highest mean depth of coverage across the different base depth thresholds evaluated (5 SNPs at >90× mean depth of coverage across the genome, increasing to 37 SNPs at 30–40×). Based on this, thresholds of 4–8× depth at a base provided SNP calls across the different genome coverage depths that were closest to the predicted true number of SNPs (Fig. 2). At thresholds of both 4× and 6× for a base, the same number of SNPs were identified at the highest genome depth of 92× and at ~40× depth.

To determine how SNP calling varied depending on the mapping reference used, we repeated the mapping of the 43 positive MRSA control runs, together with three study isolates that were ~100 SNPs from the MRSA control, to a CC30 reference (Fig. S4). These three study isolates were used to assess a similar SNP difference to the CC22 reference method (~100 SNPs), since the MRSA control is too distant (thousands of SNPs) from the CC30 reference to perform an accurate comparison of the methods. A similar pattern was observed, with the number of SNPs decreasing as the depth required to call a SNP increased (Fig. S4). As with the CC22 reference, the lowest range of SNPs called occurred between 4–8× (12–15, 11–12 and 11–12 SNPs for the three isolates, respectively).

### Defining the limit for variation in SNPs allowed for the positive control

Our methodology for sequencing does not include PhiX [8], and instead uses a positive MRSA control (MRSA MPROS0386) to control for the accuracy of base calling by the sequencer. We first determined the limit of variability in SNPs permitted for this control using data that we have generated previously for MRSA MPROS0386 over nine independent sequencing runs [8]. We calculated the expected number of SNPs compared to the mapping reference with mobile elements removed. Based on a minimum cut-off of 4× to call a SNP, a mean of 115 SNPs and range of 113–117 SNPs was identified, with a standard deviation of 1.4. Allowing 2 or 3 standard deviations from the mean resulted in cut-offs of 112.6–118.3 and 111.2–119.7 SNPs, rounded to 113–118 and 111–120 SNPs, both of which were sufficient to include all SNP differences identified across the nine sequence runs. We then applied this cut-off to the 43 positive MRSA control sequences generated here, which resulted in 111–118 SNPs and 3.2 standard deviations from the mean but fell within the rounded cut-off for 3 standard deviations from the mean designated as 111–120 SNPs. We re-calculated the number of SNPs for the positive control based on the higher cut-offs for calling a SNP of 6× and 8×. This resulted in a mean of 115 and 114 SNPs, range of 111–117 and 109–117 SNPs and standard deviation of 1.9 and 2.4 for 6× and 8×, respectively. Using 3 standard deviations from the mean, this resulted in a range of 109.1–120.3 and 106.5–120.6 SNPs (rounded to 109–120 and 107–121 SNPs), respectively. A total of 42/43 positive control run results from the study resided within these bounds, with a range of 111–116 SNPs, the exception being a single positive control isolate (ID: SAU051118), which had 108 SNPs.

### Defining a cut-off for the detection of *mec* genes

**Fig. 3.**
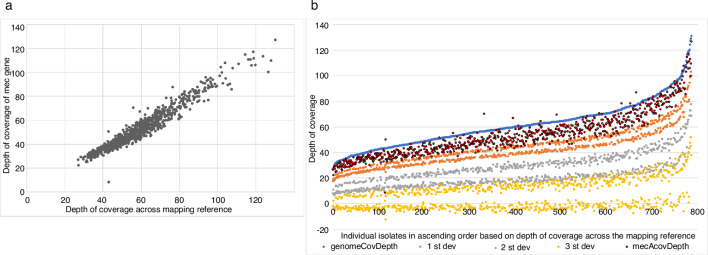
Defining a cut-off for the detection of *mec* genes. (a) Comparison of the depth of coverage across the mapping reference to the depth of coverage identified across *mecA* or *mecC* for each isolate in the study collection (*n*=786). Red dot indicates an obvious outlier. (b) Graph showing the depth of coverage across the mapping reference and across *mec* genes for the 786 study isolates, in comparison to potential QC cut-offs of 1, 2 or 3 standard deviations from the mean depth across the mapping reference.

The mean depth of coverage across the *mec* genes (*mecA* and *mecC*) in the 786 isolates was 53× (range 8×–126×). Comparison between the depth of coverage across the entire mapping reference and the depth of coverage of the *mec* genes revealed a positive association, with one clear outlier (Fig. 3a). Low depth of coverage of the *mec* gene when compared to the depth of coverage across the mapping reference could indicate MRSA contamination of an MSSA isolate. Therefore, we aimed to determine a minimum depth at which the *mec* gene could be classified as present based on 1, 2 or 3 standard deviations below the mean depth across the genome (Fig. 3b). Based on the study dataset (which had a minimum of 27× depth of coverage across the mapping reference), the lowest cut-off at 1, 2 and 3 standard deviations from the mean was 17.3×, 5.9× and −12.7×, respectively (Fig. 3b). Based on these cut-offs, a total of 21 (2.7 %), 1 (0.1 %) and 0 (0 %) of the full study collection, respectively, failed based on *mec* gene depth of coverage. Since at 3 standard deviations the *mec* gene depth would frequently pass as present at ~0× depth (Fig. 3b), this option was excluded. The single isolate failing the 2 standard deviations cut-off had a *mecA* gene depth of 8.3×, which was 20 % of the mapping reference depth (43×). In addition, the gene was split into two sections based on Ariba, one of 1192 bp and one of 849 bp. The additional 20 isolates failing QC at 1 standard deviation had *mec* gene depths 70–76 % of the depth of the mapping reference depth (30–62× over *mecA*, 41–81× over the mapping reference). Based on these data we suggest that *mec* genes with a depth that is at most 2 standard deviations below the mean depth across the mapping reference should be called as present, and those with a greater deviation should be failed and investigated further in the laboratory. This requires calculating the depth of coverage across the mapping reference, the standard deviation of depth, and the depth of coverage across the *mec* gene for each new isolate and performing a simple calculation.

### Defining a cut-off for same-species contamination

**Fig. 4.**
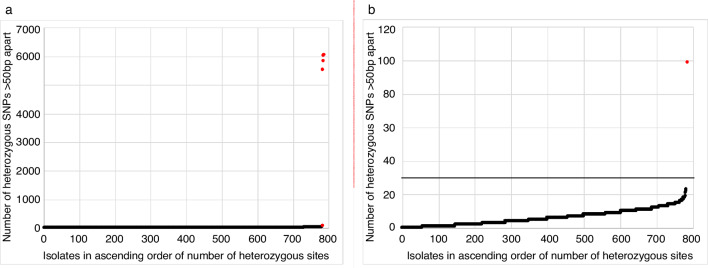
Defining a cut-off for same-species contamination. (a) Graph showing the number of heterozygous sites >50 bp apart for each of the 786 study isolates. Outliers are highlighted in red. (b) Magnification of Fig. 4a showing only those isolates with <100 heterozygous sites >50 bp apart. The line indicates the suggested cut-off of 30 heterozygous sites.

We determined a cut-off that could be used to identify same-species contamination based on the number of heterozygous sites (hetSNPs) detected when mapped to the CC22 reference genome. The number of heterozygous sites identified in the 786 clinical MRSA isolates ranged from 0 to 6046 (median 5, Fig. 4a). Stratification by ST revealed that ST22 and related STs had substantially lower numbers of heterozygous sites compared to non-ST22s (Fig. S5a). Analysis of the chromosomal location of heterozygous sites within and between STs using three representative isolates from each of the top three STs revealed that the majority of hetSNPs were clustered together in the genome, with similar ‘hot-spot’ locations identified within STs and different ‘hot-spot’ locations between STs (Fig. S6). These ‘hot-spots’ could be due to homologs as opposed to contamination, therefore to remove these hot-spots we compared the number of heterozygous sites that were identified at differing intervals of >50 bp to >500 bp apart for multiple sequences of two ST22 isolates (MPROS1839 and MPROS2264) that had been spiked with between 0–20 % of a distinct ST22 isolate (MPROS0386, see Methods). In the pure isolates (0 % contamination), the majority of hetSNPs were found to be <50 bp apart (Fig. S7). Therefore, hetSNPs <50 bp apart were removed from the analysis. Applying this filter resulted in the number of hetSNPs >50 bp apart increasing with the proportion of contamination (Fig. S7) and reduced the disparity between ST22 and other STs (Fig. S5b). Identification of hetSNPs that were 50 bp apart showed that the majority of isolates (781/786, 99.4 %) contained less than 30 hetSNPs, with five outliers containing between 99 and 6056 hetSNPs (Fig. 4a). One of these outliers was the single isolate that failed the 2 standard deviations cut-off for *mecA* gene depth, described above, further supporting that this sample was contaminated. Based on these findings, we propose that all isolate genomes should contain less than 30 hetSNPs each of which are >50 bp apart (Fig. 4b).

### Final cut-offs and application in the clinical setting

**Fig. 5.**
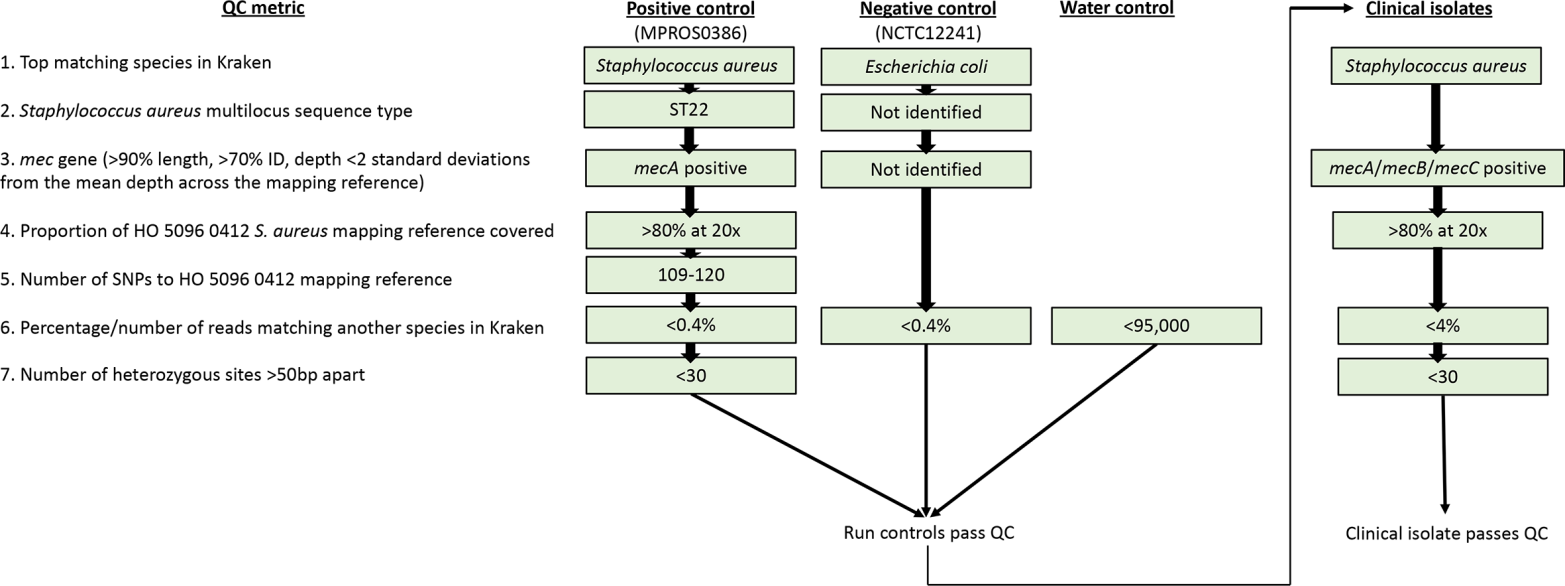
A final QC flowchart for passing/failing positive (MPROS0386), negative (NCTC12241) and no template controls and clinical isolates during clinical MRSA sequencing. Note that 0.4 and 4 % of reads matching another species in Kraken equates to contamination of 1 and 10 %, respectively. If any of the controls fail any of the QC metrics, the entire sequence run will fail and require re-sequencing. If clinical isolates fail any of the QC metrics, that single isolate is failed and should be repeated without further analysis.

The final metrics required for the controls and clinical isolates to pass QC are shown in the flowchart in Fig. 5. Based on these criteria, a total of 781/786 isolates passed all QC metrics.

## Discussion

We undertook a detailed technical evaluation of almost 800 clinical MRSA isolates to define quality metrics that support reproducible clinical MRSA sequencing. Our first question was to determine whether a single ‘generic’ mapping reference could be used to determine core genome SNPs, rather than using CC-specific mapping references [11, 13, 14]. This simplifies the informatics analysis and reduced the time for analysis because the initial *in silico* MLST followed by selection of a specific reference is not required. This technique has been used previously [15, 16], but its accuracy has not been determined. On the basis that previous studies in our setting demonstrated a predominance of MRSA CC22 [5] and further supported by a prevalence of 47 % for ST22 in this study, we mapped to a CC22 reference and compared this with a CC-specific mapping reference for all STs containing more than ten isolates. There was a clear association between the number of pairwise SNPs called by the two methods, but a higher number were called using the generic CC22 reference compared with CC-specific mapping for non-CC22 isolates. This is predictable based on the greater genetic variation between isolates assigned to different rather than the same CC. We further evaluated this using a pairwise SNP cut-off for likely transmission of 25 SNPs. This demonstrated a high concordance, suggesting that in this setting a generic reference is acceptable for clinical use. However, further work is required to determine whether this holds true in settings where ST22 is not the dominant ST and where STs with low concordance between CC22 and CC-specific mapping, such as ST36, have a higher prevalence. This will also be important to determine for cases where there is a significant shift in the dominant lineage over time. We found a reduction in prevalence of ST22 in our setting from 68 % in 2012–2013 [5] to 47 % in this study, which could alter the expected concordance of the data. Methods to overcome this will need to be developed to future-proof the technology. In the future, possible solutions for this could include a scale of SNP cut-offs dependent upon the proportion of the mapping reference covered or use of a core genome shared across STs. Until such evidence becomes available, the use of a single mapping reference and SNP cut-off for transmission remains the most computationally simple option, with no requirement for an initial MLST identification step.

In this study we used a single mapper and variant caller, but we note that there are multiple mapper and variant caller combinations available which vary in their sensitivity to SNP detection [17] and have a range of variables that can be altered. Ideally in the future, a single analysis approach would be adopted to allow between-hospital comparison. Until such time, alternative mapper/variant caller combinations will need to be tested to verify their concordance, for which the data available in this manuscript can be used as a guide.

In our current pipeline, we aim for a median depth greater than 50× based on 21 isolates and three controls [8], which was achieved in 72 % of the sequences in this study. Minor pipetting errors and low-yield runs by the sequencer can lead to lower depth, and our QC cut-off for depth is 20×. Previous studies evaluating the development of clinical MRSA sequencing have used cut-offs of 30× [18] and 60× [19]. However, the latter was suggested based on *E. coli* and *S. typhimurium* genomes and there was insufficient data to support this cut-off for *S. aureus* since there were 0 SNPs between the validation sample and reference at all coverage depths. We found that when the depth to call a SNP as present was increased towards the cut-off for the genome depth (20× in this study) the number of SNPs called fell. Our findings that the same number of SNPs were identified at the highest depth of coverage across the genome (92×) and at ~40× depth, for both the 4× and 6× thresholds, supports targeting 50× coverage across the genome and suggests that the requirement for at least 60× reported previously [19] may be too stringent. We found that defining a SNP as present at 4–8× resulted in the smallest range of SNPs identified. Based on the limit for variation in SNPs allowed in the positive control, one MRSA control isolate failed QC at 6×. When this isolate was removed, 6× resulted in the smallest range of SNPs (6 SNPs at 6× vs 8 SNPs at 4×) suggesting that a threshold of 6× is sufficient to accurately call a SNP.

Our clinical MRSA sequencing methodology includes a positive MRSA control to control for the accuracy of base calling by the sequencer rather than PhiX, which led us to define the limit of variation in SNPs allowed for the positive control. We allowed 3 standard deviations from the mean, which resulted in a SNP range of 111–120 SNPs when using the 4× cut-off for defining a SNP, up to 107–121 SNPs when using the 8× cut-off. The exact range of SNPs may vary if a different mapping or SNP calling methodology is utilized by future automated analysis pipelines, but the data provided here could be utilized to reproduce the expected SNPs for these.

Looking ahead to future practice when sequencing could replace phenotypic antimicrobial susceptibility testing, we aimed to define a robust measure for the detection of *mec* genes based on depth of coverage. Low coverage of the *mec* gene in comparison to the remainder of the genome could indicate low level contamination of an MSSA with MRSA, thereby calling a false-positive. We demonstrated that a cut-off of 2 standard deviations below the average coverage depth across the mapping reference could be used to define *mec* genes as either present or potentially absent. Our findings indicate that 1 standard deviation may be too stringent, whilst 3 standard deviations could allow false-positives. We suggest that those with *mec* genes at coverages lower than the cut-off are tested further in the laboratory to confirm absence of *mecA* and exclude a mixed population.

Compared with sequencing performed for research purposes using highly purified bacterial stock, sequencing from clinical plates runs an increased risk of contamination in which more than one *S. aureus* strain is inadvertently sequenced, although the likelihood of mixed carriage is low [5]. In addition, it is possible that during laboratory processing, contamination from one sample to another could occur. This could lead to erroneously high relatedness in the event that bases at heterozygous positions are excluded, which is standard practice. To address this, we determined a cut-off that could be used to identify same-species contamination based on the number of hetSNPs detected. We found that including all hetSNPs resulted in an inability to identify contamination since ST22 isolates had lower hetSNPs than other STs. Our 50 bp cut-off allowed hot-spots of hetSNPs, which appeared to be specific to STs, to be accounted for and resulted in five outlying isolates. The data in this study indicates that a cut-off of 30 hetSNPs >50 bp apart allows distinction between the majority of strains and outliers likely to be contaminated. However, whilst the parameters used should detect high-level (>10 %) contamination with a different *S. aureus* strain, low-level contamination and contamination with a genetically similar isolate may be missed. Parameters for detection of contamination with a different species have been described by us [8] and others [19], but to our knowledge identification of same-species contamination for *S. aureus* for clinical use has not been reported.

The final metrics required for the controls and clinical isolates to pass QC were combined into a flowchart for ease of use. This study was performed by manually inputting command lines to run each separate bioinformatic tool, and the next step in implementing real-time whole-genome sequencing into clinical practice will be the generation of a fully automated analysis-pipeline. Work on this is currently underway, and we have recently performed a pilot study into an automated pipeline developed by Next Gen Diagnostics [10]. The parameters defined in this study can be integrated into these automated pipelines, ultimately resulting in rapid hands-off sequencing and analysis in the clinical laboratory, and to guide future adopters of whole-genome sequencing. In conclusion, these methods support reliable and reproducible clinical MRSA sequencing in clinical practice.

## Data Bibliography

1. Toleman, M.S., Reuter, S., Jamrozy, D., Wilson, H.J., Blane, B., Harrison, E.M., Coll, F., Hope, R.J., Kearns, A., Parkhill, J., Peacock, S.J., Torok, M.E. *European Nucleotide Archive*, ERP005128 (2019).

2. Tosas Auguet, O., Stabler, R.A., Betley, J., Preston, M.D., Dhaliwal, M., Gaunt, M., Ioannou, A., Desai, N., Karadag, T., Batra, R., Otter, J.A., Marbach, H., Clark, T.G., Edgeworth, J.D. *European Nucleotide Archive*, PRJEB11177 (2016).

## Supplementary Data

Supplementary material 1

Supplementary material 2
