# Supplementary material for: Defining metrics for whole-genome sequence analysis of MRSA in clinical practice
Source: Microb Genom. 2020 Mar 31;6(4):e000354. doi: 10.1099/mgen.0.000354 (PMC7276698; doi:10.1099/mgen.0.000354)
Supplement: Supplementary material 1 [file mgen-6-354-s001.pdf]

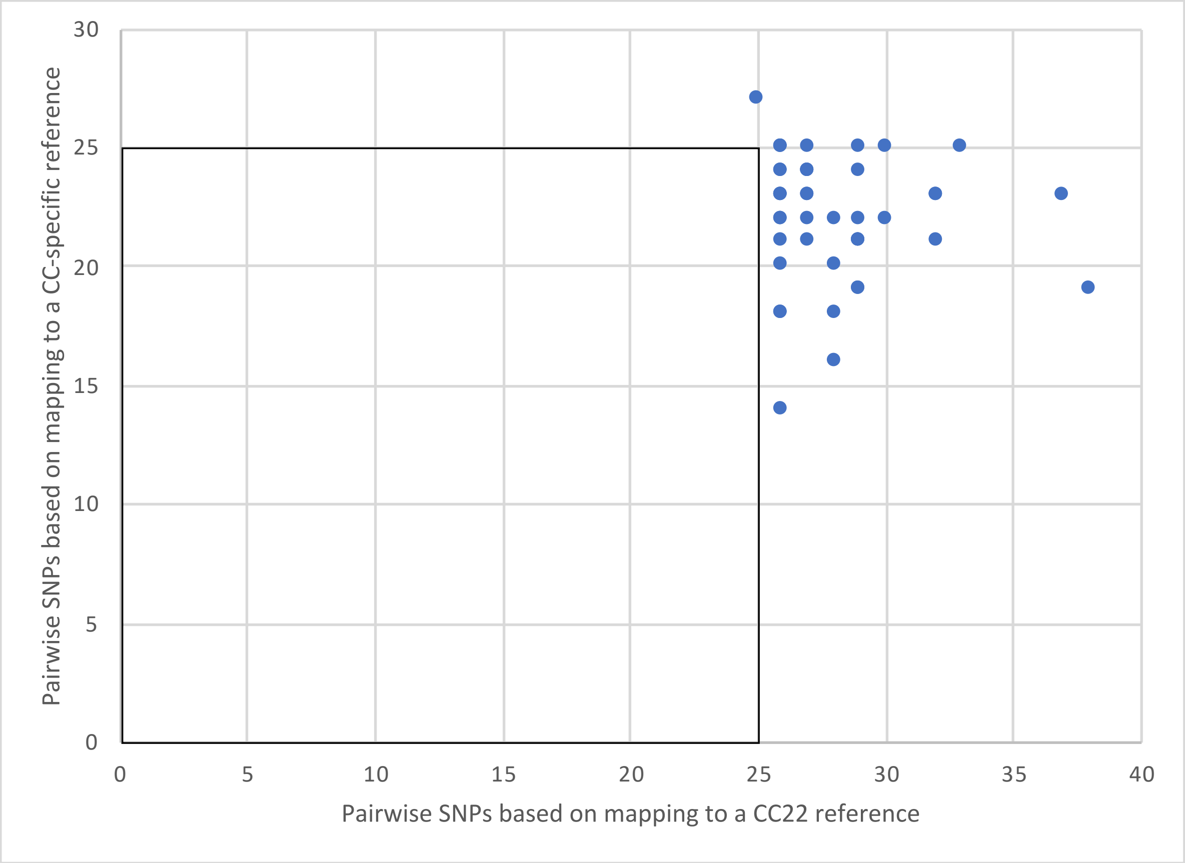

Supplementary Figure 1. Isolate pairs that were discordant in category assignment between CC-specific and CC22 mapping based on categories of 0-25 and 26-50 SNPs pairwise difference. The box indicates the differentiation between these two categories.

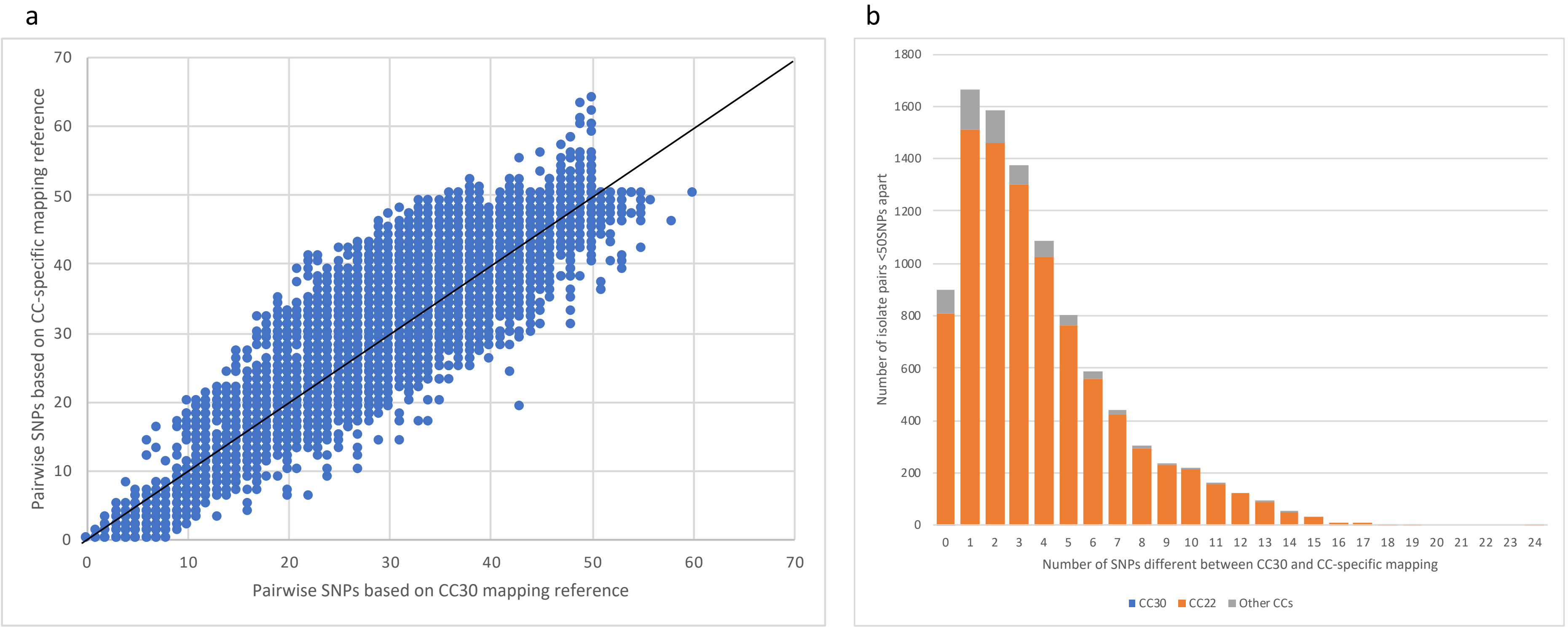

Supplementary Figure 2. Clonal complex-specific versus single mapping reference. (a) Graph showing the relationship between the pairwise SNP distances based on mapping to a CC30 reference and mapping to a CC-specific reference, for all isolate pairs <50 SNPs apart based on either method. Line indicates an exact match between the two methods. (b) Graph showing the number of SNPs different between CC30 and CC-specific mapping based on isolate pairs belonging to CC22 (orange), CC30 (blue) or other CC (grey) STs.

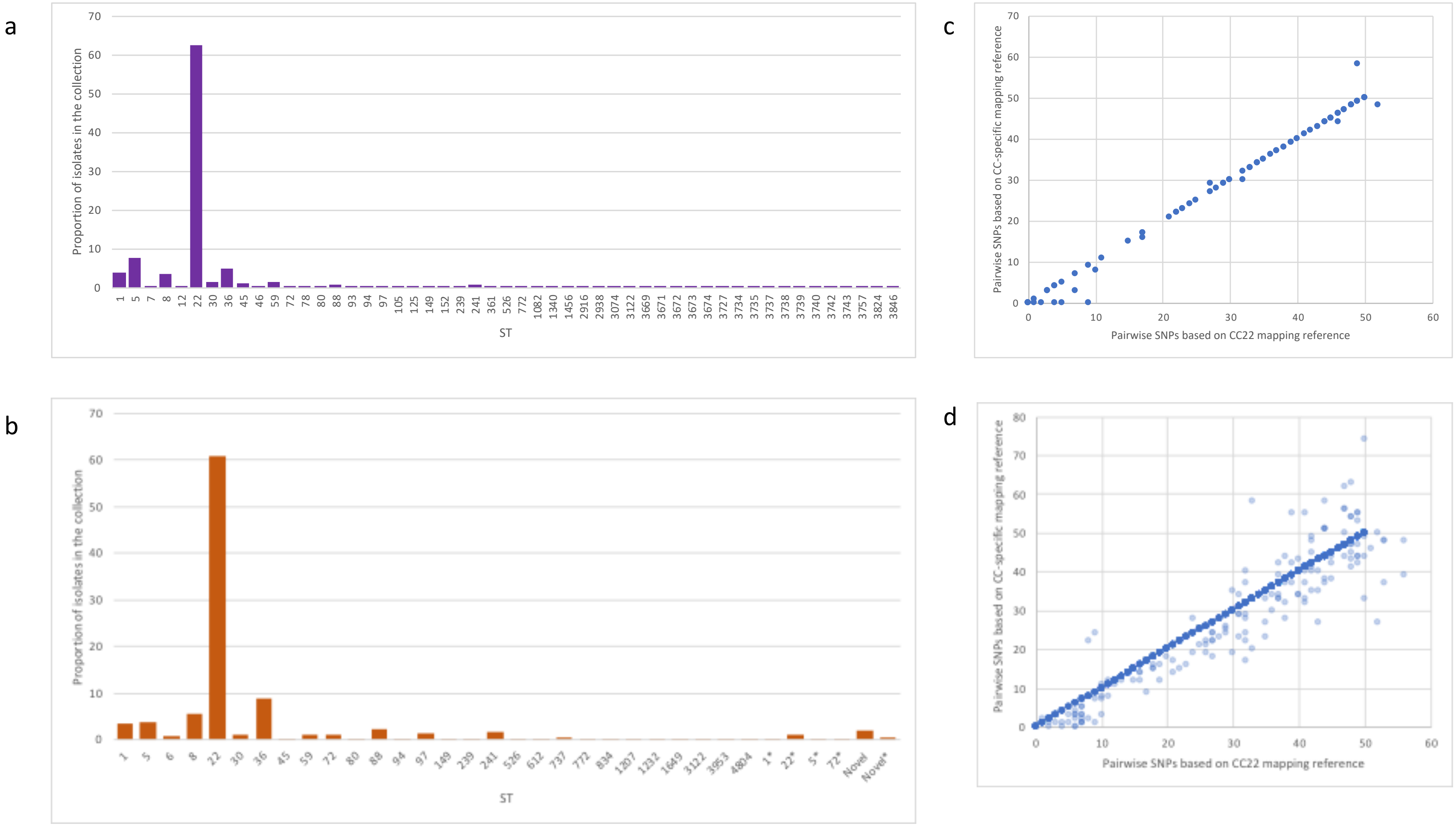

Supplementary Figure 3. Applicability of CC22 mapping to alternative clinical settings. (a) and (b): Graphs showing the distribution of STs in the national (a) and regional (b) collections. C and D: Graphs showing the relationship between pairwise SNP distances when mapped to a CC22 reference or a CC-specific reference for all isolate pairs <50 SNPs apart based on either method, that belonged to STs with >5 (national collection, c) or >10 (regional collection, d) isolates.

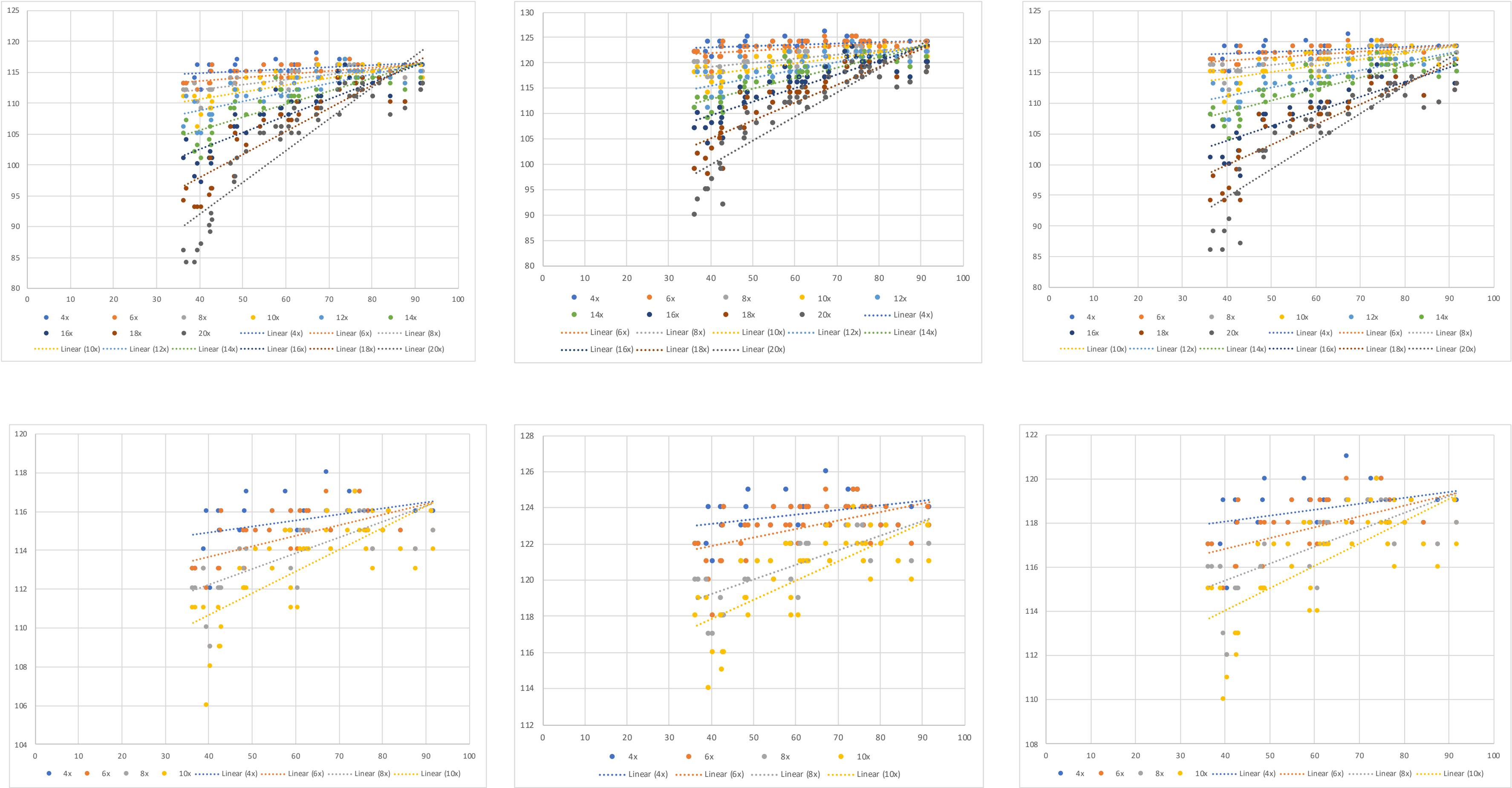

Supplementary Figure 4. Graphs show the number of SNPs identified between 43 sequence replicates of MPROS0386 and three study isolates (HICF0339, left hand side; HICF0500, centre; HICF0859, right hand side) using between 4x-20x (top row) or 4x-10x (bottom row) depth required to call a SNPs as present. X-axis indicates the depth of coverage across the MPROS0386 isolate when mapped to the CC22 mapping reference, y-axis shows the number of SNPs identified.

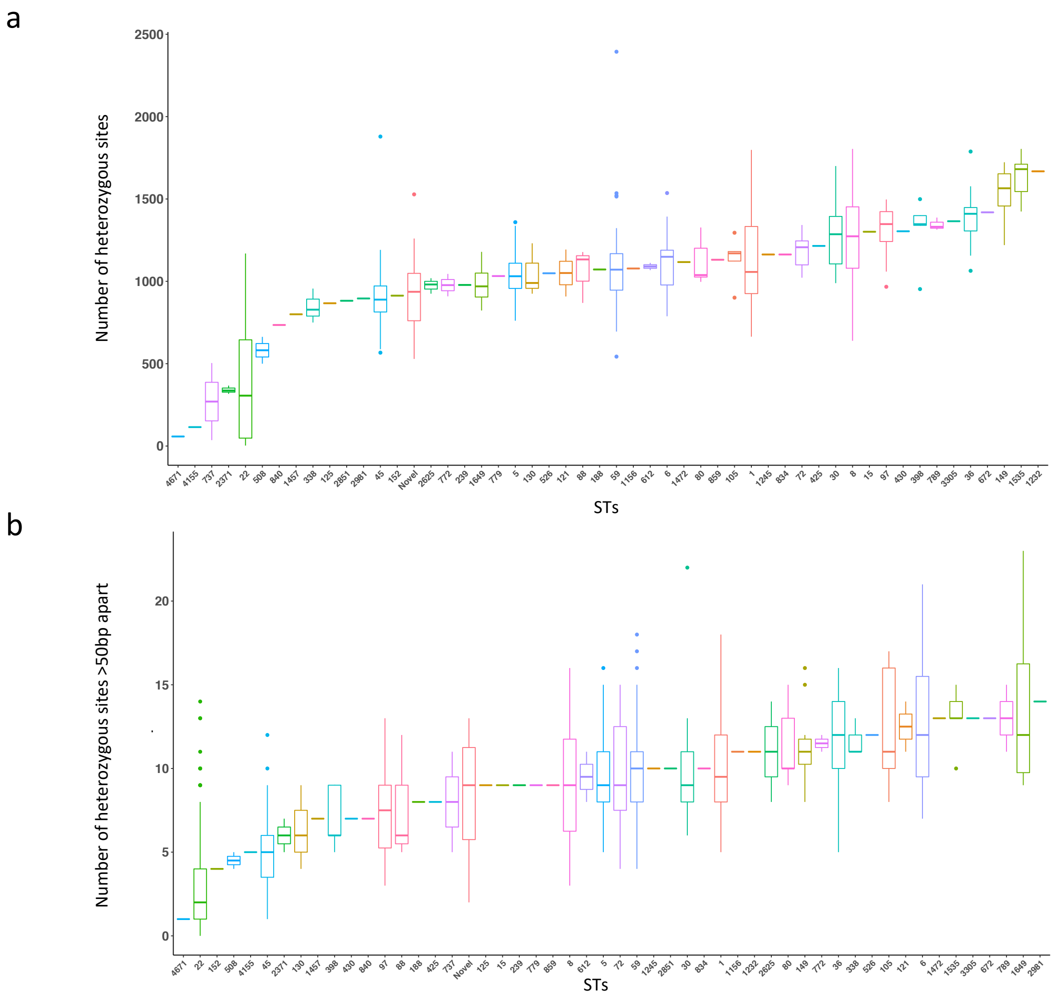

Supplementary Figure 5. Boxplot showing the distribution of heterozygous sites identified across the study collection, separated by sequence type, based on mapping to the CC22 mapping reference with MGEs removed. (a) total number of heterozygous sites, (b) number of heterozygous sites >50bp apart.

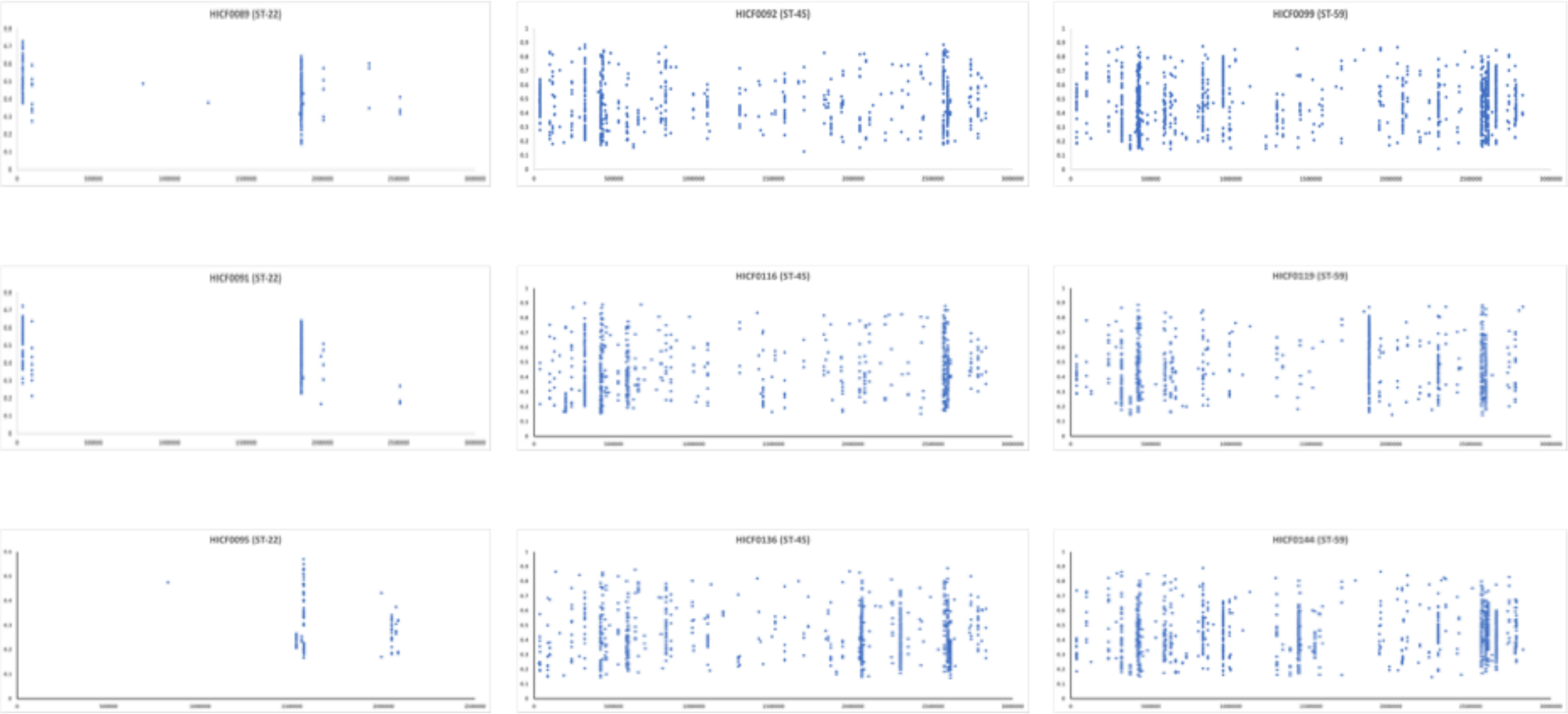

Supplementary Figure 6. Graphs show the location of heterozygous sites in three samples from each of the three most prevalent STs in the collection (ST22, left hand side; ST45, centre; ST59, right hand side). X-axis shows the location in the genome when mapped to the CC22 mapping reference, y-axis shows the proportion of the reads that support the SNP.

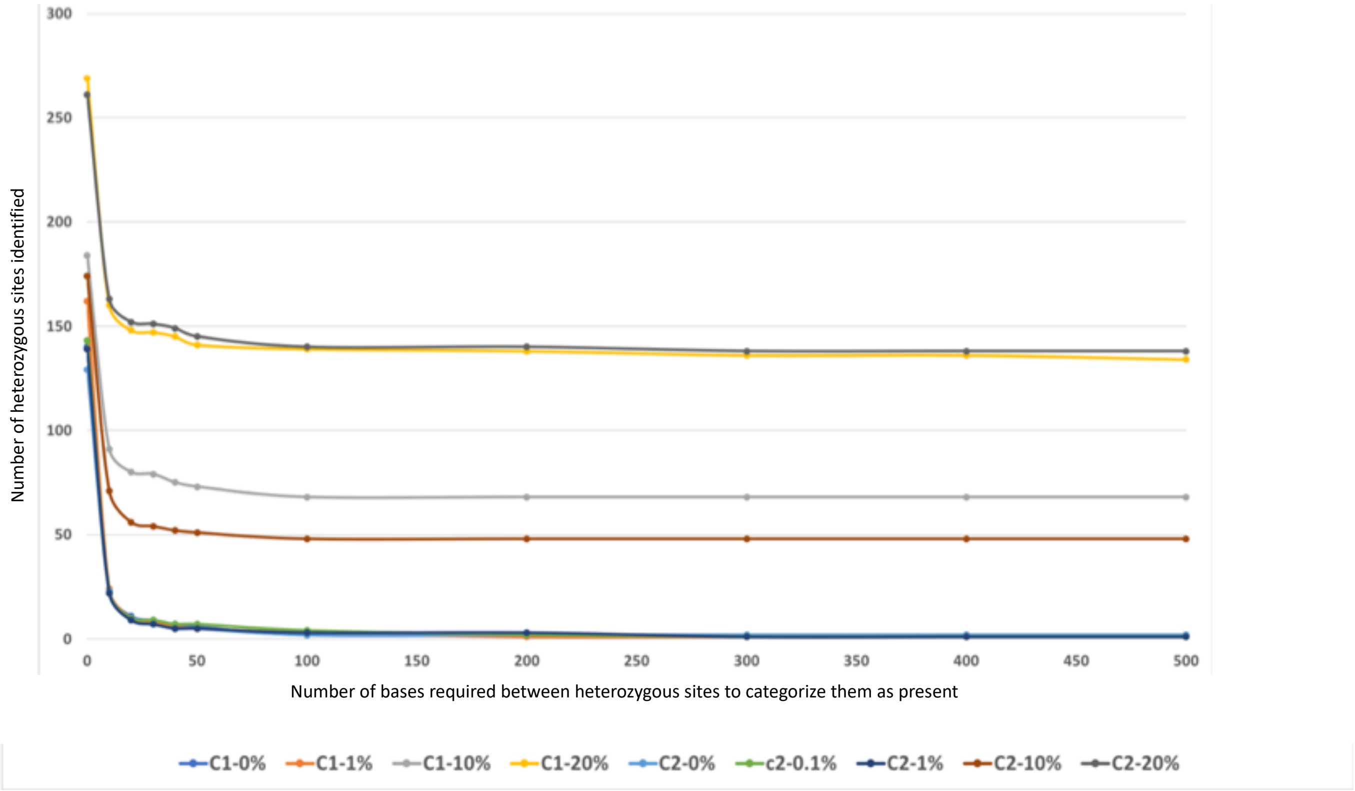

Supplementary Figure 7. Graph showing the number of heterozygous SNPs identified at different levels of contamination with another *S. aureus* strain. C1 = MPROS1839 contaminated with MPROS0386, C2 = MPROS2264 contaminated with MPROS0386, percentages indicate the percentage of MPROS0386 that each of the strains was contaminated with.

Reference	Comparator strain		4x	6x	8x	10x	12x	14x	16x	18x	20x
CC22	HO 5096 0412	Mean	115	114	113	112	110	109	106	104	101
		Range	8 (111-118)	9 (108-116)	9 (108-116)	12 (105-116)	14 (103-116)	18 (98-115)	22 (94-115)	26 (88-113)	38 (75-112)
CC30	HICF0339	Mean	112	110	107	106	105	103	101	99	96
		Range	16 (106-121)	13 (105-117)	13 (102-114)	15 (99-113)	17 (112-96)	20 (93-112)	26 (87-112)	29 (82-110)	35 (74-108)
	HICF0500	Mean	113	111	110	109	108	107	104	101	97
		Range	12 (109-120)	13 (107-119)	13 (105-117)	13 (103-115)	18 (98-115)	19 (96-114)	22 (92-113)	27 (84-110)	31 (78-108)
	HICF0859	Mean	111	110	109	108	105	102	97	95	91
		Range	12 (106-117)	13 (105-117)	13 (103-115)	14 (102-115)	17 (96-112)	20 (92-111)	23 (85-107)	25 (81-105)	31 (72-102)

Supplementary Table 3. Table showing the mean and range of SNPs identified across the 43 sequence replicates of MPROS0386 based on different read depths required to call a SNP as present.
